# Differences in brain circuitry for appetitive and reactive aggression as revealed by realistic auditory scripts

**DOI:** 10.3389/fnbeh.2014.00425

**Published:** 2014-12-09

**Authors:** James K. Moran, Roland Weierstall, Thomas Elbert

**Affiliations:** Department of Psychology, University of KonstanzKonstanz, Germany

**Keywords:** aggression, violence, magnetoencephalography, oscillatory brain activity, motivation

## Abstract

Aggressive behavior is thought to divide into two motivational elements: The first being a self-defensively motivated aggression against threat and a second, hedonically motivated “appetitive” aggression. Appetitive aggression is the less understood of the two, often only researched within abnormal psychology. Our approach is to understand it as a universal and adaptive response, and examine the functional neural activity of ordinary men (*N* = 50) presented with an imaginative listening task involving a murderer describing a kill. We manipulated motivational context in a between-subjects design to evoke appetitive or reactive aggression, against a neutral control, measuring activity with Magnetoencephalography (MEG). Results show differences in left frontal regions in delta (2–5 Hz) and alpha band (8–12 Hz) for aggressive conditions and right parietal delta activity differentiating appetitive and reactive aggression. These results validate the distinction of reward-driven appetitive aggression from reactive aggression in ordinary populations at the level of functional neural brain circuitry.

## Introduction

Aggression has long been regarded as a dichotomous phenomenon (Weinshenker and Siegel, 2002; Mcellistrem, 2004; Meloy, 2006), belonging to two different motivational systems: One representing self-defense with the avoidance of threat and danger, and the other broadly representing the planning and execution of proactive attacks.

Reactive aggression is relatively well defined both behaviorally and in neurobiological terms as a functional response to threat. It is part of the freeze-flight-fight-fright-faint sequence of the defense cascade (Weinshenker and Siegel, 2002; Mcellistrem, 2004; Meloy, 2006; Schauer and Elbert, 2010), and can be observed in humans and animals alike. Its function is defense against perceived threat, and it is distinguished by high arousal, and emotions of negative valence, like anger, frustration, and fear. Animal studies suggest that the underlying neural circuitry of reactive aggression is largely the same in predators (e.g., cats) and prey (e.g., rats): The activity in the medial amygdala, the hypothalamic attack area, and the periaqueductal gray axis, determines aggressive behavior in animals. The activity of this axis is regulated by other areas, in particular the prefrontal cortex, as well as the lateral septum, and the brain stem monoaminergic nuclei (Gregg and Siegel, 2001). The orbitofrontal cortex (OFC) plays a role in evaluating rewards vs. expectations of rewards, and as such is associated with frustration (Blair, 2004), as well as recognizing violations of social norms (Blair and Cipolotti, 2000), both of which can be a trigger for reactive aggression.

When interviewing soldiers, ex-combatants, and child soldiers who have committed violent acts, many report adverse consequences of traumatic stress, frequently with problems in emotion regulation and a risk for engaging in reactive aggressive behavior (Marsee, 2008). However, a contrary narrative also frequently emerges, in that a significant proportion of ex-combatants report fighting and killing not as being frightening but rather as being exhilarating, and pleasurable (Elbert et al., 2010). Killing, though often experienced as extremely stressful at first, becomes easier and finally even enjoyable to many fighters (Maclure and Denov, 2006; Elbert et al., 2010). We denote this as appetitive aggression, which we conceptualize as fundamentally hedonic in character and related to reinforcing qualities of the violent act itself. This includes, for example, the thrill of hunting your prey, pulling the trigger and hitting the target, the cries of the victim, and the scent of blood. This is as opposed to instrumental aggression, which defines rewarding qualities of violence as those that follow as a result of violence, such as increased social status, power, and money. Our field observations of appetitive aggression in post-conflict lands show that it is widespread in the general population, and not limited to a psychopathic minority. In support of our observations, other theorists have also recognized the necessity of understanding aggression in terms of normal rather than abnormal psychology (Nell, 2006; Neitzel and Welzer, 2011; Kröber, 2012). Furthermore, this aggression arises in a variety of cultural contexts, e.g., the Democratic Republic of the Congo (Hecker et al., 2012), Uganda (Weierstall et al., 2012b), Colombia (Weierstall et al., 2013), and has been acknowledged by German WWII veterans more than 60 years after their deployment (Weierstall et al., 2012a).

Neuropsychological research examining aggression has conceptualized a complement to reactive aggression, variously labeled “proactive,” “predatory” or “instrumental” aggression (Meloy, 2006). These areas of research, viewing this form of aggression as a fundamentally dysfunctional psychopathology have focused upon forensic populations of ASD, psychopathy (Raine et al., 1998; Blair, 2007; Yang and Raine, 2009) and conduct disorder (CD) (Marsh et al., 2013), and other developmental disorders (Dodge et al., 1997; Vitiello and Stoff, 1997; Jones et al., 2009). However, neuroimaging investigations are faced with an interpretive challenge in that these disorders also feature high reactive aggression (Yang and Raine, 2009). Other neural differences are focused on diminished capacity for aversive conditioning, insensitivity to social anger cues (Blair, 2004, 2010), and impaired emotional empathy (Blair, 2013). The hedonic and rewarding element of this form of aggression is less often emphasized. We believe this is important, as it is apparently something that can be awakened in the general population, and is functional and adaptive in certain circumstances, such as war (Elbert et al., 2010). Its expression is not linked to any necessary neurobiological deficit, such as empathy or aversive conditioning mentioned above. In certain animals there is a functional predatory form of aggression complementary to reactive aggression (Panksepp and Zellner, 2004). These distinctions have been localized in subcortical systems. For example in cats, there are two distinct aggressive behaviors: One is a defensive posture marked by hissing, piloerection, and a second predatory hunting mode. These two forms of aggression can be linked to mediobasal and lateral parts of the hypothalamus respectively and higher afferent structures (Flynn et al., 1970). Electrical stimulation of the periaqueductal gray, for instance, can elicit aggressive behavior in the cat, but not in the rat (Shaikh et al., 1987). Separate areas within the hypothalamus for predatory vs. reactive aggression can be similarly distinguished in other animals, including mice (Lin et al., 2011), and rats (Tulogdi et al., 2010; Toth et al., 2012; see Haller, 2013 for review). Though this evidence is compelling in animal models, humans have not shown this neat differentiation of aggression subtypes in these subcortical regions, showing only a hypothalamically mediated general aggression (Weiger and Bear, 1988).

Though a cat has a simple neurobiological switch between two discrete forms of aggression, human appetitive and reactive aggression can display significant overlap. An untried soldier can simultaneously feel terror and exhilaration in battle (Elbert et al., 2010). Nell (2006), observing the predatory hunting behavior of chimpanzees notes that two behaviors can switch back and forth, between the fear of confronting the defensive prey in one moment to the exhilaration of conquering it. In humans, punishing someone for a transgression can evoke a feeling of reward. The latter instance is demonstrated in the Taylor Aggression paradigm, where reactive elements of aggression are operationalized in terms of punishment with provocation, reactive aggression activates reward-related subcortical areas such as the ventral striatum (De Quervain et al., 2004; Krämer et al., 2007).

Though some theorists emphasize the applicability of animal models to human aggression (Panksepp and Zellner, 2004), it is likely that humans have evolved on a different evolutionary trajectory from that of other predators, like cats and have developed different forms of inhibition for intra-species killing. Indeed, with our complex societies, killing conspecifics will have a variety of complex consequences, advantages, disadvantages, relating ultimately to survival, accrual of resources and reproductive success. These strategies will have likely been subject to natural and sexual selective pressure (Duntley and Buss, 2011). The complexity of the social use of violence suggests that again activity in cortical regions, particularly the prefrontal cortex, is decisive for modulating, inhibiting or allowing positive or exciting feelings relating to violence, whether toward humans or animals. For example, the OFC plays a role in evaluating social cues, and thus control whether expression of violence is appropriate or not. Indeed lesions in this region often lead to aggressive behavior in humans, as shown, for example, in Vietnam veterans (Grafman et al., 1996). This suggests, in concert with studies in primates (Machado and Bachevalier, 2006), that in humans, a more highly developed cortical response provides inhibitory function over subcortical circuits for both forms of aggression. To see it from the perspective of psychopathy research: Psychopaths have major deficits in cortical connectivity, producing either a want of empathy or punishment insensitivity (Blair, 2007, 2013), but this failure in inhibition does not in itself explain the existence of the violent impulses themselves. Psychopaths also show functional abnormalities in reward-structures such as the Ventral Striatum (VS) (Carré et al., 2013) and in dopamine release in the Nucleus Accumbens (Buckholtz et al., 2010). As argued above by research in conflict lands, hedonically motivated violence is not restricted to a psychopathic minority, and functional cortical activity in appetitive aggression should be observable in ordinary people.

It should be possible to evoke appetitive aggression and differentiate it from reactive aggression in the laboratory with non-clinical participants. Although many studies have found ways to provoke aggression in the laboratory, systematically separating appetitive and reactive components has not been attempted before. Violent computer games provide a socially sanctioned means of enjoying aggression, mimicking both the fear and excitement of combat. One neuroimaging experiment using violent computer games as a stimulus found OFC, amygdala, and anterior cingulate cortex (ACC) activity, but did not separate subtypes (Weber et al., 2006), another study from the same group did choose reward and defensive areas as a priori regions of interest, but interpreted results in terms of reactive aggression and empathy deficits (Mathiak and Weber, 2006). A PET study from Koepp et al. (1998) showed dopaminergic activation in response to video game playing, indicating that the violent gaming activated reward related systems in the brain. Perach-Barzilay et al. (2013) operationalized reactive and proactive aggression through a financial decision task (Social Orientation Paradigm), and used inhibitory rTMS in low frequency theta bands on left and right dorsolateral prefrontal (DLPFC) areas to modulate it. Although they predicted frontal left and right lateralized activity corresponding to approach/withdrawal motivations, respectively (Davidson, 1992 and Harmon-Jones et al., 2010), they found a uniformly left frontal lateralization for both aggression subtypes.

Another technique to induce aggression is to use dramatic testimonies, where participants are asked to put themselves in the position of a killer vs. victim (Weierstall et al., 2014). This has been shown to increase or decrease testosterone in men, according to whether the person is assigned to take the appetitive (perpetrator) or reactive (victim), role in the story, respectively. The purpose of the present experiment was to induce appetitive and reactive aggression in non-clinical participants in the same manner, measuring functional neural activity with MEG. In detail, we present exactly the same auditory testimony of an alleged murderer, describing a murder to three different between-subjects groups, prefaced by a different motive, either appetitive (lust, exhilaration, hunting, excitement), reactive (fear, anger, frustration, self-defense) or a motivationally neutral control. We analyze the spontaneous activity during the murder story itself, rather than the prior emotional prime. Even though there are no perceptual or signal differences between groups, we predict differences in oscillatory activity unique to each form of aggression. Earlier studies have used imagination scenarios to successfully activate aggression related areas (Pietrini et al., 2000), however, our experiment has the additional advantage of presenting a realistic and complex stimulus to each group which is perceptually identical, whilst at the same time separating reactive from appetitive aggressive responses. This use of complex real-world stimuli has become more popular with the advance of a variety of different analytical techniques (Hasson et al., 2004; Wilson et al., 2008), and provides us with the opportunity to improve the comparability between field and laboratory research for validating phenomena identified in a natural context.

We predict a differential oscillatory activity between reactive and appetitive and control conditions, since this is exploratory, we examine activity from low to high frequency bands across the whole brain.

## Materials and methods

### Participants

Participants were 61 male students, to make 3 between-subjects conditions of approximately 20 participants each. From this, 11 people were removed prior to analysis, due to the presence of non-physiological of artifacts in the MEG data (appetitive, *N* = 17, reactive, *N* = 14, and control, *N* = 19). The age ranged between 20 and 39 years.

Participants were selected to allow MEG-recording free from non-physiological artifacts (no ferromagnetic materials). Screening ascertained that no clinical or neurological disorders were present. Random group allocation to one of three experimental groups for the selected volunteers was decided before meeting the participant. There were no significant differences in age, or handedness as measured by the Edinburgh Handedness inventory (Oldfield, 1971) between these groups.

Participants received 25€ for participation, or course credit in the 2.5 h-long investigation. The University of Konstanz ethical review board approved the study and the experiment was carried out in accordance with the declaration of Helsinki. All participants gave written informed consent.

### Stimulus materials and design

#### Independent stimuli

The experiment used a story narrated from an audio recording by a male professional actor posing as an alleged murderer. This story is told in two parts in each condition. In the first part, he describes the motives and emotions that preceded a murder committed by him, and in the second part, he describes in graphic detail the way he murdered another man (4 min, 59 s long). The second part of the story is written in such a way that it can be used across all three conditions and the difference between the three between-subjects conditions is entirely in the first part, covering all levels of vivid experiencing, namely emotions, cognitions, sensations and behavior. The first and second parts together are presented to the participant as one seamless recording, although only the MEG activity during the second, perceptually identical part is analyzed. Our outcome measure is the brain activity during the actual description of the murder itself.

#### Preface stories

There were three different stories prefacing the murder. In the *reactive condition* (4 min, 10 s long), the murderer describes his lifelong feelings of rage, anger, and frustration at being treated badly by others, thus the murder is more interpretable as an act of reactive aggression. In the *appetitive condition* (preface duration: 4 min, 11 s long), he describes his lifelong enjoyment of violently attacking people, e.g., in childhood gangs and as a football hooligan, describing feelings of excitement and happiness. In this condition, the murder is intended to be interpreted as an act of appetitive aggression. In the control condition there is a background description of the lead-up to the murder, but no indication of the emotional state of the murderer (duration: 1 min, 10 s). Additionally, all participants heard a second story (duration: 5 min, 9 s, order of presentation counterbalanced), detailing the daily life of an ordinary student (waking up, catching the bus, reading a book, waiting for an appointment). This is intended to provide an intra-subject baseline condition.

#### Coverstory

The instructions given to the participant whilst listening to the stories were given to encourage the participant to empathize with the murderer: Participants were told that the experiment was to see how well laypeople could perform the job of a criminal profiler and that a good criminal profiler can empathize with the criminal. This conceit was deemed necessary to circumvent any potential strong resistance to admitting to socially undesirable aggression in ordinary German students. Reimagining an aggressive point of view in a criminal profiling context should be familiar from popular media, in which the main character occupying the socially desirable role of detective or forensic psychologist often has an uncanny empathy with the mind of the criminal. All participants received a debriefing on the study goals after the end of the experiment.

#### Subjective ratings

A variety of questionnaires were administered immediately after the presentation of the stories. These assessed certain facets of the participant themselves as well as aspects of their reaction to the murderer in the role of profiler. Participants were asked to rate their own feelings of appetitive aggression in their capacity of profiler whilst listening to the alleged murderer with Weierstall and Elbert's (2011) *Appetitive Aggression Scale* (AAS). Reactive aggression was measured with Buss and Perry's (1992) *Aggression Questionnaire* (AQ). Current mood was rated with the *Positive and Negative Affect Schedule* (PANAS) (Watson et al., 1988), a 10 positive and 10 negative adjectives. On 5-point-Likert scales subjects rated the plausibility of the story and their identification with the protagonist and perpetrator. See Supplementary Material for further details of these questionnaires.

#### Procedure

Participants were randomly assigned to one of the three different conditions. All underwent two MEG measures: An experimental and a baseline measure. Written instructions regarding the role of the participant as “profiler” were read to participants before each measure. The order of experimental and baseline measures was counterbalanced. Standard instructions were read to each participant before each story.

For the experimental conditions, participants were told that they were to hear the testimony of an accused murderer, and that their task as profiler was to put themselves in the position of the murderer, to see, feel everything from his point of view, and to put aside moral scruples they might ordinarily have about a murderer. They were told that their reactions afterwards to being put in this position would be assessed with questionnaires. For the neutral story, participants were similarly told to put themselves in the position of the person they were hearing. After hearing the story, participants were asked to complete the questionnaires.

#### Apparatus and physiological data collection

***MEG***. Participants were in a prone position whilst MEG signals were measured. The MEG was a 148 channel whole-head magnetometer (MAGNES™ 2500 WH. 4D Neuroimaging, San Diego, USA). Before the MEG measures, head reference points (nasion, left and right ear, and overall headshape) were digitized with a Polhemus 3Space® Fasttrack. The neuromagnetic signals were digitized with a sampling rate of 678.17 Hz, and bandpass filtered from 0.1 to 200 Hz. Noise reduction was carried out offline, using distant reference sensors. The data was pre-processed using FieldTrip scripts (Oostenveld et al., 2011). The data was high and low pass filtered from 2 to 40 Hz, with a 14–18 Notch filter, to remove noise produced by a nearby train line. The continuous activity of the experimental and baseline conditions were cut into 150 epochs, which were 2 s long. Subsequently, epochs with large jumps were removed as well as 4 channels that were consistently noisy across subjects. An Independent Components Analysis (ICA) was then carried out to identify components associated with eye movement, muscle and heartbeat related artifacts. There was no difference across groups in the number of epochs deleted within experimental and baseline conditions. The experimental component had significantly more epochs than baseline [*F*_(1, 47)_ = 12.89, *p* = 0.001]. There was however, no interaction effect across groups. The order of presentation of the stories did not have an influence upon the number of removed epochs, and the difference in remaining epochs was small (both >140).

Single-trial averaging of the epochs was carried out by means of a Hanning taper, followed by a Fast-Fourier-Transformation. The epochs were averaged to obtain the mean power-spectra for each frequency, in intervals of 0.5 Hz. Since the epochs were not evoked, there was no time dimension factored into these calculations. This was carried out for both experimental and baseline conditions. Thereafter, data for both the experimental and baseline conditions were normalized by dividing the difference in the experimental and neutral condition by the Standard Deviation of the neutral condition. This created a standardized power difference (SPD). The following frequency bands were chosen for analysis: Delta (2–5 Hz), Theta (5–8 Hz), Alpha (8–12 Hz), and Beta/Gamma (20–40 Hz) Each of the three conditions were compared with a *t*-test for independent samples (Matthews and Altman, 1996; Cubillo et al., 2012) on each individual sensor. For the resultant map of *t*-values, criteria for a cluster indicating significant differences between group conditions were set at a minimum of 5 contiguous electrodes (Doñamayor et al., 2012).

## Results

### Validity of experimental manipulation

There were no differences in rated morality of the stories across conditions [*F*_(2, 48)_ = 1.63, *p* = 0.21]. The stories in each condition were rated as plausible, with a total mean of 3.67 on a Likert scale of 1–5. There were no significant differences between groups [*F*_(2, 48)_ = 0.68, *p* = 0.51].

Participants in all three groups identified more strongly with the perpetrator than victim [*F*_(1, 47)_ = 14.64, *p* < 0.001]. One-Way ANOVAs showed that there were no differences in appetitive vs. reactive ratings for the underlying motivation in the aggressor across the three groups as measured by the AAS [*F*_(2, 47)_ = 1.14, *p* = 0.33] and AQ [*F*_(2, 47)_ = 0.61, *p* = 0.85]. There was a significant group effect for PANAS ratings of the positivity of the story [*F*_(2, 46)_ = 4.95, *p* = 0.01], in comparison to the neutral story, with people in the appetitive group [*M* = 10.82, *SE* = 2.11] having a significantly more positive rating of the story than those in the reactive group [*M* = 2.31, *SE* = 1.99, *p* = 0.003]. Furthermore, AAS scores interacted with group ratings of positive arousal during the story, with higher AAS scores predicting significantly greater positive arousal in the appetitive group (*r* = 0.63, *p* = 0.007), but not reactive (*r* = 0.18, *p* = 0.55) or control groups (*r* = 0.16, *p* = 0.53). All above analyses fulfilled assumptions for their respective parametric tests, and were replicated with the 11 excluded participants from the final MEG sample.

### MEG results

Clusters indicating significant group differences were found for Delta (2–5 Hz) and Alpha Activity (8–12 Hz) differentiating the three conditions.

#### Delta range

**Figure 1:**
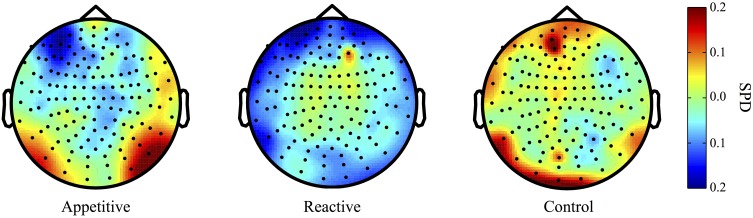
**Delta Band (2–5 Hz) topographical plots showing differences in SPD each experimental group**. The circle represents the whole-head MEG layout for appetitive (*N* = 17), reactive (*N* = 14), and control (*N* = 19) conditions.

Both the appetitive and reactive conditions had frontal negative delta clusters that differentiated them from the control condition (see Figure 1 for plots of SPDs).

**Figure 2:**
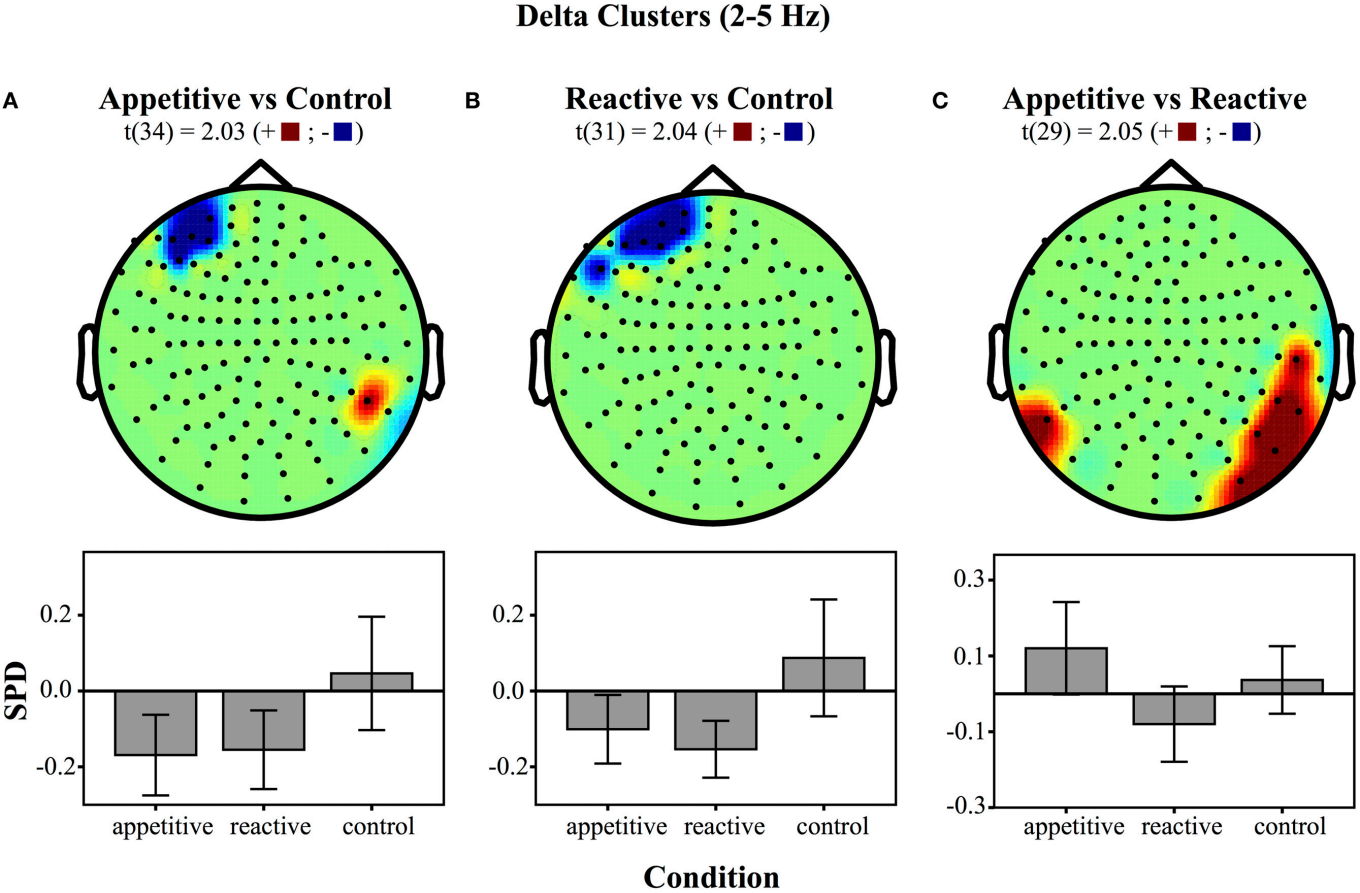
**Clusters drawn from critical *t*-values (for *p* < 0.05) in the Delta band (2–5 Hz) for comparisons between the three experimental conditions (above) and bar graphs of differences within clusters (below), (error bars represent 95% CI): **(A)** Appetitive vs. Control **(B)** Reactive vs. Control and **(C)** Appetitive vs. Reactive**.

***Left Frontal Clusters***. A cluster in the left frontal region was found for the appetitive vs. control, there was a general significant difference across conditions [*F*_(2, 49)_ = 4.28, *p* = 0.020], follow up Bonferroni corrected comparisons showed significant differences between appetitive (*M* = −0.17, *SE* = 0.05) and control (*M* = 0.05, *SE* = 0.07, *p* = 0.035), but not between reactive (*M* = −0.15, *SE* = 0.05) and control (*p* = 0.073) or appetitive and reactive (See Figure 2A).

A left frontal delta ROI was found for comparison of reactive vs. control. A comparison of the SPD between conditions was significant [*F*_(2, 49)_ = 5.06, *p* = 0.010], follow up Bonferroni corrected comparisons showed significant differences between reactive (*M* = −0.15, *SE* = 0.03) and control (*M* = 0.09, *SE* = 0.07, *p* = 0.016); appetitive (*M* = −0.10, *SE* = 0.04) was not significantly different from control (*p* = 0.059), or reactive conditions (See Figure 2B).

***Right Parietal/Temporal Cluster***. For the delta activity in the right parietal/temporal region, there was an overall difference between conditions [*F*_(2, 49)_ = 3.81, *p* = 0.029], follow-up Bonferroni contrasts showed a difference between appetitive (*M* = 0.12, *SE* = 0.06) and reactive (*M* = −0.08, *SE* = 0.05, *p* = 0.025) but not for the control condition (*M* = 0.04, *SE* = 0.04, *p* = 0.658); the reactive vs. control comparison was also non-significant (*p* = 0.320) (See Figure 2C).

#### Alpha range

**Figure 3:**
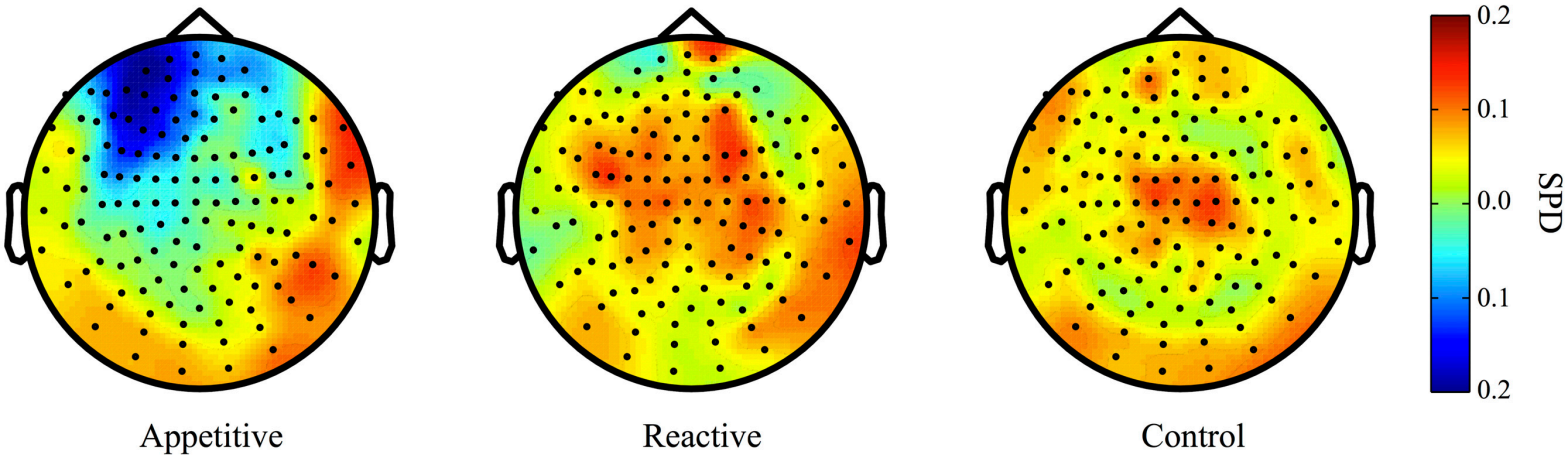
**Alpha Band (8–12 Hz) topographical plots showing SPD for each experimental group**. The circle represents the whole-head MEG layout for appetitive (*N* = 17), reactive (*N* = 14), and control (*N* = 19) conditions.

**Figure 4:**
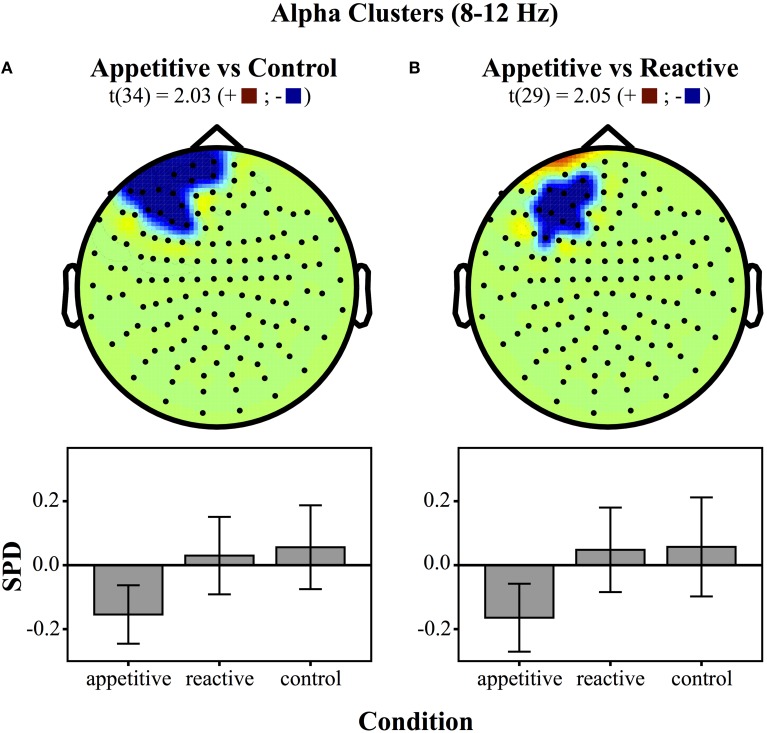
**Clusters drawn from critical *t*-values (for *p* < 0.05) in the Alpha band (8–12 Hz) for comparisons between the three experimental conditions (above) and bar graphs of differences within clusters (below), (error bars represent 95% CI): **(A)** Appetitive vs. Control **(B)** Appetitive vs. Reactive**.

For comparisons between appetitive and control in the alpha range (8–12 Hz) (see Figure 3 for plots of SDPs), there was a left frontal cluster indicating significant differences between conditions [*F*_(2, 49)_ = 4.04, *p* = 0.018]. Bonferroni contrasts showed that appetitive (*M* = −0.15, *SE* = 0.04) was significantly more negative than control (*M* = 0.06, *SE* = 0.06, *p* = 0.023), but not reactive groups (*M* = 0.03, *SE* = 0.06, *p* = 0.087), there was no difference between reactive and control (See Figure 4A).

The cluster distinguishing alpha from reactive showed a generally significant differences across groups [*F*_(2, 49)_ = 3.87, *p* = 0.028]. Bonferroni contrasts showed that appetitive (*M* = −0.16, *SE* = 0.05) was significantly more negative than control (*M* = 0.06, *SE* = 0.07, *p* = 0.044), but not more negative than reactive (*M* = 0.05, *SE* = 0.06, *p* = 0.089). There was no difference between reactive and control (See Figure 4B).

#### AAS scale correlations

**Figure 5:**
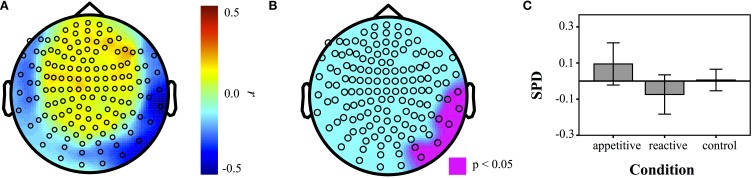
**Correlation maps of SPD with AAS measures for each sensor across entire sample (*N* = 50)**. **(A)** Correlation coefficients at each sensor, **(B)** map of sensors that were significant at *p* < 0.05. **(C)** Barplot of SPD for each condition in the ROI defined by AAS correlations (error bars represent 95% CI).

To supplement the above group analyses, correlations of the AAS scale with power in each individual sensor for different frequency bands were made across the entire sample (*N* = 50). A resultant correlation map showed a parietal/temporal cluster, which was then tested for group differences. Experimental groups differed significantly [*F*_(2, 49)_ = 3.33, *p* = 0.045]. Bonferroni contrasts showed that appetitive (*M* = 0.09, *SE* = 0.06) was significantly more positive than reactive (*M* = −0.07, *SE* = 0.05, *p* = 0.040). The control group (*M* = 0.01, *SE* = 0.03) lay between these and was not significantly different to the other two conditions (appetitive vs. control: *p* = 0.45, reactive vs. control: *p* = 0.65) (See Figure 5).

## Discussion

Is there a difference in the neural circuitry underlying reactive and appetitive aggression? This experiment sought to induce both aggressive modes in a sample of non-clinical participants, using realistic stimuli depicting the two forms of aggression and asking them to imagine themselves as protagonists in these scenarios. Inducing identification in participants for the perpetrator was successful, in that all participants identified more with the perpetrator than with the victim, the story was regarded as plausible, and there were no potentially confounding differences in the perceived morality of the story across conditions.

Although the grouping did not influence scores on questionnaire measures of appetitive or reactive aggression, it is possible that the scales are more sensitive to trait rather than state qualities of aggression. Indeed, the appetitive group produced a stronger positive arousal state than the reactive group, and AAS scores moderated this effect, with people higher in appetitive aggression (trait) responding to the appetitive condition with more positive arousal (state).

The MEG results showed a low frequency left frontal cluster in the delta band (2–5 Hz) for both appetitive and reactive conditions, in comparison to control conditions. In the alpha band (8–12 Hz) the appetitive group showed a left frontal cluster differentiating it markedly from the control group, with reactive aggression in between these two. Reactive and appetitive conditions were differentiated from each other in the delta band in a right parietal/temporal region. Moreover, a follow-up test showed that trait-level appetitive aggression was correlated with a cluster of sensors in the same region. Within this appetitive aggression-related region, the appetitive and reactive conditions showed opposite patterns of synchronization and desynchronization, respectively.

These results show that left frontal activity is associated with aggression generally, rather than separating subtypes. Its specific left localization is congruent with findings from other researchers, who also note a frontal left bias in aggression processing. Adolescents with Conduct disorder also show structural deficits in specifically left amygdala activity (Sterzer et al., 2007). Perach-Barzilay et al. (2013) used a decision-making task where participants could punish confederates either as reactive response to provocation, or as a planned “proactive” aggression, inhibiting theta activity by means of continuous transcranial magnetic stimulation (cTMS) for left vs. right frontal regions, potentiated aggression only in the left DLPFC. A metaanalysis of ASD noted predominantly left DLPFC deficits were associated with impulsivity-related violence. However, right OFC and ACC activity was also a characteristic feature of these disorders (Yang and Raine, 2009). The alpha negativity in the left region for the appetitive condition is in accord with studies associating aggression with left-mediated approach motivation (Coan and Allen, 2004; Hermans et al., 2008; Harmon-Jones et al., 2010; Van Honk et al., 2010). In this motivational picture of the brain, left frontal brain activity (operationalized as alpha desynchronization) is associated with approach motivation, and right activity with fear and withdrawal-related activity.

The area where clear differences between appetitive and reactive aggression were found was in the right parietal/temporal regions in the delta band, distinguishing both induced state appetitive aggression, as well as trait appetitive aggression. This region is one of the candidate regions sensitive to fetal testosterone. Comparing men and women, researchers found a correlation between gray matter volume in this region and fetal exposure to testosterone in men only (Lombardo et al., 2012). This region is associated with theory of mind, and empathy with others (Saxe and Powell, 2006; Decety and Lamm, 2007). Although all conditions involved seeing oneself in the position of a perpetrator, only the appetitive condition showed a specific synchronization, this together with the association with scores on our appetitive aggression scale suggests a specific association with appetitive aggression. Disinhibiting subcortically mediated reward systems relating to violence could be related to reduction in empathy for others. The aggression of Psychopaths is frequently attributed to empathy deficits, most often stemming from frontal subcortical and cortical deficits (Blair, 2007, 2013), but also in a similar right parietal/temporal region (Müller et al., 2008a,b). The implication is, that when this empathy is out of play, rewarding aspects of violence find expression. A lack of emotional empathy is assumed to be a primary factor in the cruelty of a psychopath (Blair, 2013). However, other neuroimaging studies have shown abnormal processing in reward-related regions of the psychopathic brain such as the VS (Carré et al., 2013) and Nucleus Accumbens dopamine release (Buckholtz et al., 2010). We cannot say whether it is weaker empathy regions that fail to restrain reward-related processes, or abnormal reward processes that overwhelm empathy regions. The latter interpretation would imply that for a psychopath, a lifetime of cruelty to others, motivated by a lust to hurt, produces empathy-related deficits as a consequence, viz. empathy related processing would be generally inactive, leading across the course of development to the atrophy in these regions. This one study needs replication before making any stronger claims, but it is congruent with the idea that aggression is a very dynamic process, in that non-clinical participants show cortical changes relating to appetitive aggression. It also matches with the idea that psychopathy is dimensional in character (Hare and Neumann, 2008). It is perhaps comparable to the real-life situation of training ordinary men to work as soldiers, empathy is suppressed externally, to reduce soldiers' inhibitions about killing the enemy, e.g., by using dehumanization techniques, such as giving them animal names like “rats” or “monkeys” (Grossman, 1996; Staub, 2006).

We were able to demonstrate a change in the affective state of people in this laboratory scenario, operating with the assumption that a story about a violent man will evoke emotions associated with violence. We emphasize that in this imaginative act, parallel emotional circuits become activated (Blair, 2005; Decety and Meyer, 2008) though these need not implicate active behavior. Some limitations intrinsic to this form of research with naturalistic stimuli need to be acknowledged. Our aggressive story was perceptually identical across all three conditions, and thus differences in the signal between groups cannot be attributed to any differences in auditory perception or processing. One could object however, that the different ideas mentioned in the first part of the experiment could have lead to different imagined scenarios for each group that go beyond forms of aggression. We hold that the large sample size within each group, and the fact that the effects are the product of single-trial averaging of a large number of trials, means that the differences between groups can be meaningfully interpreted as relating to correlates of different aggression forms. A future proof of differential neural processing (e.g., different media; evoked rather than spontaneous stimuli) would certainly be necessary to elaborate the differences between these two forms of aggression. An interesting validation of our technique could be to test for neural activity on ex-combatants high in appetitive aggression retelling their own stories. It is a frequent observation that remembrances of violence evoke powerful reliving experiences, either negative, as in the case of PTSD, or positive, in the case of appetitive aggression (Elbert et al., 2010). In this way the laboratory model of evoking appetitive aggression in ordinary people via stories would be compared to the imaginative re-experiencing of the phenomenon in itself. One further issue that requires examination in the future is the relationship between appetitive and reactive aggression. Some experimental observations suggest overlap of the hedonic qualities with reactive responses (Krämer et al., 2007) and field observations suggest that one “acquires at taste” for battle, moving from fear and revulsion to euphoria and power (Elbert et al., 2010). Studying this process of change from reactive to appetitive in the laboratory within one person would give us more insight into the dynamics of the processes involved.

The results generally speak for the possibility that a hedonic, positive response to aggression is an intrinsic general part of an individual's behavior, and is observable at the level of functional neuroactivity, suggesting that it is fundamentally separate to systems relating to reactive aggression.

## Author contributions

James K. Moran, Roland Weierstall, and Thomas Elbert designed research; Thomas Elbert acquired the funding James K. Moran performed research; James K. Moran and Roland Weierstall analyzed data; and all three authors wrote the paper.

### Conflict of interest statement

The authors declare that the research was conducted in the absence of any commercial or financial relationships that could be construed as a potential conflict of interest.
