# Supplementary material for: Differences in brain circuitry for appetitive and reactive aggression as revealed by realistic auditory scripts
Source: Front Behav Neurosci. 2014 Dec 9;8:425. doi: 10.3389/fnbeh.2014.00425 (PMC4260506; doi:10.3389/fnbeh.2014.00425)
Supplement: Supplementary file 1 [file DataSheet1.DOCX]

1. **Supplementary Material**

## Methods

## Appetitive Aggression Scale (Weierstall and Elbert, 2011)

A 15-item questionnaire measuring hedonic aspects of aggressive acts. Items consists of statements about feelings towards aggression, to which the person responds on a 5-point Likert scale, from 0 (‘disagree’) to 4 (‘agree’). The questionnaire has been tested on a large number of different populations (including soldiers and ex-combatants) in different cultures (Weierstall and Elbert, 2011; Weierstall et al., 2011; Weierstall et al., 2012; Hecker et al., 2013). It has shown good psychometric properties (Weierstall and Elbert, 2011). Participants were asked to answer from the point of view of their feelings in the role of profiler as they heard and empathized with the story. The AAS is accompanied by a 15-item questionnaire asking participants about their own experiences of violence in their lives.

## Buss & Perry Aggression Questionnaire (Buss and Perry, 1992)

A 27-item questionnaire measuring four different dimensions of aggression: Physical and verbal aggression, hostility, and anger. As most of the items in this questionnaire are related to aggressive behavior motivated by negative emotions and thus encompass reactive aggressive behavior. This questionnaire was administered to capture reactive elements of aggression, not covered by the appetitive scale (German version: Herzberg, 2003)). All items are scored on a 4-point Likert scale ranging from 1 (‘disagree’) to 4 (‘agree’) and were summed up to a total score.

## Positive and Negative Affect Schedule (Watson et al., 1988)

A 20-item list of adjectives, 10 positive and 10 negative, participants rated the extent to which their mood reflected each of these words on a 5 point Likert scale from 1 ‘very slightly or not at all’ to 5 ‘extremely’. Participants completed this survey for both experimental and baseline conditions, to provide a basal measure of how they felt whilst listening to the stories. This is a widely used scale with good psychometric properties, in both German (Krohne et al., 1996; Mearns et al., 2008), and English versions (Crawford and Henry, 2004).

## Participant Identification with Protagonist and Perpetrator

Participants were asked to indicate the extent to which they identified with perpetrator or victim on a 5-point likert scale, with answers ranging from ‘0’ (‘Not at all’) to ‘4’ (‘Extremely’). This has been used in a previous study (Weierstall et al., 2014). This was a control for identification with the perpetrator rather than the victim.

## Morality

Since morality has been shown to have neural correlates, differences between rated morality of the stories could affect the measured signal (Moll et al., 2005). Participants rated the morality of the experimental story by placing a stroke on a visual analogue scale from 0 to 10 cm, which was converted to a score from 0 (morally defensible) to 100 (morally reprehensible).

## Plausibility of Story

Participants rated the plausibility of the story on a 5-point likert scale with answers ranging from 0 ‘Not at all’ to 4 ‘Extremely’.

1. **References (Supplementary Material)**

Buss, A. H., and Perry, M. (1992). The Aggression Questionnaire. J. Pers. Soc. Psychol. 63, 452-459.

Crawford, J. R., and Henry, J. D. (2004). The Positive and Negative Affect Schedule (PANAS): Construct validity, measurement properties and normative data in a large non-clinical sample. *Brit. J. Clin. Psychol.* 43, 245-265.

Hecker, T., Hermenau, K., Maedl, A., Hinkel, H., Schauer, M., and Elbert, T. (2013). Does perpetrating violence damage mental health? Differences between forcibly recruited and voluntary combatants in DR Congo. *J. Trauma. Stress* 26, 142-148. doi: 10.1002/jts.21770.

Herzberg, P.Y. (2003). Faktorstruktur, Gütekriterien und Konstruktvalidität der deutschen Übersetzung des Aggressionsfragebogens von Buss und Perry. [Factor structure, validity criteria and construct validity of the German translation of the Aggression Questionnaire from Buss and Perry]. *Zeitschrift für Differentielle und Diagnostische Psychologie* 24, 311-323.

Krohne, H. W., Egloff, B., Kohlmann, C., and Tausch, A. (1996). Untersuchungen einer deutscher Fassung der "Positive and Negative Affect Schedule" (PANAS). [Investigation of a German version of the “Positive and Negative Affect Schedule”]. *Diagnostica* 42, 139-156.

Mearns, J., Catanzaro, S. J., Schwarz, T., Pfeiffer, N., and Backenstrass, M. (2008). Reliabilität und Validität der deutschsprachigen Version der Generalized Expectancies for Negative Mood Regulation (NMR) Scale. [Reliability and validity of the German language version of the Generalized Expectancies for Negative Mood Regulation (NMR) Scale]. *Diagnostica* 54, 43-51. doi: 10.1026/0012-1924.54.1.43.

Moll, J., Zahn, R., De Oliveira-Souza, R., Krueger, F., and Grafman, J. (2005). The neural basis of human moral cognition. *Nature Reviews: Neuroscience* 6, 799-809.

Watson, D., Clark, L. A., and Tellegen, A. (1988). Development and measurement of brief measures of positive and negative affect: The PANAS scales. J. Pers. Soc. Psychol. 54, 1063-1070.

Weierstall, R., and Elbert, T. (2011). The Appetitive Aggression Scale-development of an instrument for the assessment of human's attraction to violence. *Eur. J Psychotraumato.l* *2*, doi: 10.3402/ejpt.v2i0.8430.

Weierstall, R., Moran, J., Giebel, G., and Elbert, T. (2014). Testosterone reactivity and identification with a perpetrator or a victim in a story are associated with attraction to violence-related cues. *Int. J. Law Psych.* 37, 304-312.

Weierstall, R., Schaal, S., Schalinski, I., Dusingizemungu, J. P., and Elbert, T. (2011). The thrill of being violent as an antidote to posttraumatic stress disorder in Rwandese genocide perpetrators. *Eur. J. Psychotraumatol.* 2, doi: 10.3402/ejpt.v2i0.6345.

Weierstall, R., Schalinski, I., Crombach, A., Hecker, T., and Elbert, T. (2012). When combat prevents PTSD symptoms--results from a survey with former child soldiers in Northern Uganda. *BMC Psychiatry* 12, 41. doi: 10.1186/1471-244X-12-41.
